# Development of Individualized Minimally Invasive Therapy and Multidisciplinary Care for Gastrointestinal Cancer

**DOI:** 10.31662/jmaj.2022-0082

**Published:** 2022-06-24

**Authors:** Yuko Kitagawa

**Affiliations:** 1 Department of Surgery, Keio University School of Medicine, Tokyo, Japan

**Keywords:** gastroenterological cancer, minimally invasive treatment, multi-modal treatment, individualization

## Abstract

Today, approximately one in every two people in Japan is diagnosed with cancer at least once in their lifetime, making cancer control a matter of national concern. However, advances in diagnosis and treatment have made it possible to cure 70%-80% of cancers. Early-stage malignancies can now be resolved in nearly all cases, with the current challenge lying in how to fully cure the patient in a manner that conserves the affected organ and its function. Minimally invasive, function-preserving surgery for early-stage tumors will likely further evolve with the introduction of robotics and individualized surgery. Meanwhile, cancers that are difficult to control―advanced or intractable forms―require a multidisciplinary approach combining diverse therapeutic strategies. The future of treatment for advanced cancer promises to bring innovations such as individualized treatment planning based on cancer genome analysis, precise determination of treatment responsiveness using gene mutations as indicators, and early diagnosis of metastasis/recurrence through liquid biopsy. This paper provides an overview of the current state and future prospects of multidisciplinary cancer care, with a focus on the author’s field of expertise―upper gastrointestinal (GI) cancers.

## I. The Concept for Standard Surgical Procedures in Radical Surgery for GI Cancer

Rudolf Virchow, a 19^th^-century pathologist, recognized the metastasis of cancer via the lymph system. The 19^th^-century surgical oncologist William Halsted developed the radical mastectomy surgery―the surgical removal of not only the breast but also the pectoralis major and minor muscles and the axillary lymph nodes―in an endeavor to control local recurrence and lymph node metastasis. In this and other ways, various standard surgical procedures necessary for improving local control through preventive lymphadenectomy with sufficient resection margins were established for many types of solid-tumor cancers.

Radical surgery for gastric cancer involving resection of at least two-thirds of the stomach and d2 lymphadenectomy was also conceived in line with this concept and contributed to the improvement of therapeutic outcomes. Standard surgical procedures involving sufficient preventive lymphadenectomy of regions with potential for lymph node micrometastasis were established for many GI cancers.

## II. Changes in GI Cancer Therapy through the Dissemination and Growth of Luminal Endoscopy and Laparoscopic Surgery

Remarkable advances in endoscopic diagnosis of gastric cancer increased the frequency of detection of early-stage malignancies, particularly mucosal cancer. This led to a shift in thinking, as surgical oncologists began seeing the across the board application of radical surgery to all cases of gastric cancer as an excessively invasive strategy. Starting in the 1970s, endoscopic surgery was developed for mucosal gastric cancer, which has a low risk of lymph node metastasis in its very early stage. However, due to the technical limitations of endoscopic surgery at that time, this approach was confined to lesions small enough to be resected in one operation, as the fractional resection of larger lesions across multiple surgeries posed a high risk of recurrence. For this reason, its use was limited to an extremely small range of cases.

In the 1990s, surgery performed with laparoscopes or thoracoscopes rapidly spread in GI surgery and many other fields. Ohgami et al. developed laparoscopic local resection of the stomach for mucosal gastric cancers that had until then been completely unresectable ^(1)^. Later, however, the use of the Ohgami technique declined following the development of endoscopic submucosal dissection in the 2000s, whereby luminal endoscopy could be used to more safely and more successfully remove lesions indicated for the Ohgami technique. The same era saw the development and rapid dissemination of a laparoscopic procedure involving gastrectomy and lymphadenectomy to treat early gastric cancers for which lymph node metastasis could not be ruled out. A large gap formed between endoscopy-indicated lesions and surgery-indicated lesions in terms of the invasiveness of the procedures used.

## III. Development of Individualized Limited Surgery Guided by Accurate Intraoperative Diagnosis of Lymph Node Metastasis

Currently, it is extremely difficult to detect microscopic lymph node micrometastases through preoperative diagnostic imaging. As one solution to this challenge, we turned our attention to the sentinel node (SN) hypothesis. This revolutionary theory postulates that a cancer can be diagnosed as non-metastatic if no metastasis is found in the SN, the first draining lymph node of the primary tumor. In the 1990s, it began to be clinically applied to cancers such as melanoma and breast cancer. However, it was thought to be difficult to apply to GI cancer, as the complex drainage of lymph in this region meant that there were many possible routes of lymph node metastasis. We developed a method for identifying the SN of GI tumors through endoscopic injection of pigment and a radionuclide-labeled colloid into the submucosa, and we reported on its validity for intraoperative diagnosis of lymph node metastasis through SN biopsy in esophageal, gastric, and colon cancer ^(2), (3), (4)^. We also conducted a prospective multicenter trial on gastric cancer and reported a very high rate of accuracy in the diagnosis of lymph node metastasis from SN biopsy of cT1N0 tumors less than 4 cm in diameter ^(5)^. In addition, we reported a novel technique combining laparoscopy and luminal endoscopy that achieved full-thickness resection of gastric cancer without exposing the gastric lumen to the peritoneal cavity, with the aim of conserving postoperative gastric function in SN-negative patients through radical minimizing of the resection size ^(6)^. Based on these results, we initiated a trial for clinically applying this individualized limited surgery guided by SN biopsy to early gastric cancer, under the Advanced Medical Care B framework ^(7)^. Patient enrollment has been completed, and we are confirming the noninferiority of this approach to the standard surgical procedure in terms of long-term outcomes.

## IV. Further Growth of Individualized Surgery and Potential for Additional Indications

In recent years, the incidence of esophagogastric junction (EGJ) tumors has been gradually increasing in Japan and other Asian countries. We conducted a prospective study on the distribution of lymph node metastases from EGJ tumors in a joint effort by the Japan Esophageal Society and the Japanese Gastric Cancer Association ^(8)^. We reported that the tumor’s esophageal invasion depth is a risk factor for mediastinal lymph node metastasis and that the extent of lymphadenectomy and the surgical approach/procedure can be individualized to a certain degree. Furthermore, through radioisotope mapping of lymph drainage, we established that the drainage direction and SN distribution in esophageal cancer (including EGJ tumors) is elusive and varies widely from patient to patient ^(9)^. We believe that diverse limited surgery approaches can be applied even to EGJ tumors based on individualized mapping of lymph drainage and the result of SN biopsy ^(10)^. In contrast with laparoscopic SN biopsy of gastric cancer, mediastinal SN biopsy of esophageal cancer is technically complicated, and it can be rather difficult to accomplish with a minimally invasive approach. Nevertheless, we expect that the development and dissemination of robot-assisted surgery in recent years will make it possible to more readily apply mediastinal SN biopsy to esophageal cancer in the future.

## V. The Limitations of Extended Surgery for Advanced/Intractable Cancer

Compared with Western nations, Japan has achieved superior short-term and long-term outcomes for GI cancer surgery across many fields. In the 1980s, Japanese GI surgical oncologists endeavored to build upon that strong track record by engaging in various forms of extended surgery intended to further enhance long-term outcomes. However, those efforts did not always succeed in reaping the expected results. Randomized multicenter trials in Japan were unable to achieve improved prognosis versus the standard surgical care for procedures such as extended lymph node and nerve plexus dissection for pancreatic cancer, gastric cancer surgery extended with preventive lymphadenectomy around the aorta, and left thoracotomy for gastric cancer with esophageal invasion. Based on these findings, Japan’s surgical community determined that it would be difficult to improve the prognosis for advanced cancer simply through extension of the range of resection and lymphadenectomy and thus began to develop perioperative adjuvant therapies.

## VI. The Role of Quality of Surgery within Multidisciplinary Care

Today, surgery alone does not suffice as the standard of care for many GI cancers, which often require some form of adjuvant therapy. In the case of esophageal cancer, neoadjuvant chemotherapy has become part of the standard strategy, and research is underway to further strengthen and optimize neoadjuvant therapy. Chemoradiotherapy before surgery is now the standard treatment for esophageal cancer in Western countries. The Japan Clinical Oncology Group, of which the author is a member, is presently conducting a randomized study (JCOG1109) comparing three preoperative therapies: chemotherapy with cisplatin plus 5-fluorouracil (CF; the current standard treatment), chemotherapy combining CF with docetaxel, and chemoradiotherapy using CF ^(11)^. Generally speaking, chemoradiotherapy has a high response rate as a preoperative treatment, but in the context of multidisciplinary care involving surgery, this does not always lead to the best long-term outcomes. It has been reported that complications stemming from intensive neoadjuvant treatment―namely, systemic inflammation, microthrombi formation, and antitumor immunity attenuation―may promote cancer metastasis and recurrence. We have examined the relationship between postoperative pneumonia incidence and prognosis in patients who received preoperative chemotherapy in a clinical trial, and we reported that postoperative pneumonia may worsen the prognosis ^(12)^. In multidisciplinary care involving surgery, it is likely that the quality of surgery in terms of lymphadenectomy precision, frequency of complications, and other such measures has a considerable impact on the outcomes. Surgeons around the world are looking to see whether the aforementioned JCOG1109 study will demonstrate the superiority of either the Western strategy of relying on chemoradiotherapy to cover for inadequate local control by surgery or the Japanese approach, which achieves very high local control with the combination of surgery and intensive systemic chemotherapy.

## VII. Transformation of the Surgeon’s “Turf” and Required Skillset by Advances in Multidisciplinary Care

The evolution of nonsurgical treatment has immensely transformed care for unresectable advanced cancers for which surgery was once not an option. In the case of locally advanced unresectable esophageal cancer, whose standard treatment has been chemoradiotherapy, we have reported that chemotherapy with a combination of three drugs made resection possible in approximately 40% of the patients studied ^(13)^. The advent of immune checkpoint inhibitors (ICIs) has also greatly reshaped multidisciplinary care for solid-tumor cancers. We conducted a phase II study that administered an ICI to patients with esophageal cancer refractory or intolerant to the standard treatment ^(14)^, and we later demonstrated significant improvement of prognosis by the ICI in second-line treatment versus the standard chemotherapy ^(15)^. Presently, we are conducting an investigator-initiated study of the safety and efficacy of a strategy combining that ICI with neoadjuvant chemotherapy ^(16)^. In addition, an international multicenter study has shown the efficacy of the same ICI as adjuvant therapy following chemoradiotherapy for esophageal cancer, demonstrating the potential for the use of ICIs in not only unresectable/recurrent cases but also perioperative therapy ^(17)^. However, given the risk for immune-related adverse events associated with ICIs, caution needs to be exercised in their adjuvant use before or after relatively highly invasive surgery, such as for esophageal cancer. Advances in various forms of nonsurgical therapy are shifting the surgeon’s role more toward the treatment of advanced cancers. This means that surgeons will need to be well-versed in chemotherapy, molecularly targeted therapy, radiotherapy, and immunotherapy and to be capable of safely performing high-level surgery and complex postoperative management following the use of those intensive preoperative treatments.

## VIII. Working toward Rational Individualized Care for Advanced/Intractable Cancers

In recent years, research has been done on a strategy for curing cancers such as esophageal and rectal malignancies with radical chemoradiotherapy, thereby avoiding the need for surgery. This strategy is predicated on early detection of recurrence and the appropriate use of salvage therapy when recurrence is found. As part of its clinical research, Keio University Hospital performs gene panel testing on all consenting patients who have undergone surgery for solid-tumor cancer. This will enable us to identify genetic mutations in the cancer cells of each patient and to perform sophisticated cell-free DNA monitoring for cancer cells in the bloodstream following treatment. In addition, we believe that it will be possible to use liquid biopsy as an indicator for determining whether or not salvage surgery is indicated for patients responding to intensive neoadjuvant therapy.

## IX. Outlook for the Future

Looking ahead to the future of GI cancer therapy, care for early-stage cancers that can be fully cured will need to focus not on applying a standard treatment across the board but on how to minimize invasiveness and preserve function. As for advanced cancers that defy complete cure, further progress must be obtained in our ability to provide high-level multidisciplinary care. In both cases, cutting-edge technologies need to be harnessed to achieve greater individualization of care. Minimally invasive, function-preserving surgery for early-stage tumors will likely further evolve with the introduction of robotics and individualized surgery that leverages forthcoming advances in imaging technology for visualizing cancer cells. Meanwhile, the future of treatment for advanced cancer promises to bring innovations such as individualized treatment planning based on cancer genome analysis, precise determination of treatment responsiveness using gene mutations as indicators, and early diagnosis of metastasis/recurrence through liquid biopsy.

## Article Information

This article is based on the study, which received the Medical Award of The Japan Medical Association in 2021.

### Conflicts of Interest

＜Grants or contracts from any entity＞

Takeda Pharmaceutical Co., Ltd.　（Grants to our institution）

CHUGAI PHARMACEUTICAL CO., LTD. （Grants to our institution）

TAIHO PHARMACEUTICAL CO., LTD （Grants to our institution）

Yakult Honsha Co. Ltd. （Grants to our institution）

ASAHI KASEI PHARMA CORPORATION （Grants to our institution）

Otsuka Pharmaceutical Co., Ltd. （Grants to our institution）

ONO PHARMACEUTICAL CO., LTD. （Grants to our institution）

TSUMURA & CO. （Grants to our institution）

Kyouwa Hakkou Kirin Co., Ltd. （Grants to our institution）

DAINIPPON SUMITOMO PHARMA Co., Ltd. （Grants to our institution）

EA Pharma Co., Ltd. （Grants to our institution）

Astellas Pharma Inc. （Grants to our institution）

Toyama Chemical Co., Ltd （Grants to our institution）

MEDICON INC. （Grants to our institution）

KAKEN PHARMACEUTICAL CO. LTD. （Grants to our institution）

Eisai Co., Ltd. （Grants to our institution）

Otsuka Pharmaceutical Factory Inc. （Grants to our institution）

TEIJIN PHARMA LIMITED. （Grants to our institution）

NIHON PHARMACEUTICAL CO., LTD. （Grants to our institution）

Nippon Covidien Inc. （Grants to our institution）

　

＜Payment or honoraria for lectures, presentations, speakers bureaus, manuscript writing or educational events＞

CHUGAI PHARMACEUTICAL CO., LTD. (Personal fees)

TAIHO PHARMACEUTICAL CO., LTD (Personal fees)

ASAHI KASEI PHARMA CORPORATION (Personal fees)

Otsuka Pharmaceutical Factory Inc. (Personal fees)

SHIONOGI & CO., LTD. (Personal fees)

Nippon Covidien Inc. (Personal fees)

Ethicon, Inc. (Personal fees)

ONO PHARMACEUTICAL CO., LTD. (Personal fees)

Olympus Corporation (Personal fees)

Bristol-Myers Squibb K.K. (Personal fees)

AstraZeneca K.K. (Personal fees)

MSD K.K. (Personal fees)

Smith & Nephew K.K. (Personal fees)

KAKEN PHARMACEUTICAL CO., LTD. (Personal fees)

ASKA Pharmaceutical Co., Ltd. (Personal fees)

　

＜Other financial or non-financial interests＞

<Other financial or non-financial interests>

Ono Pharmaceutical Co., Ltd. (Personal fees)

Bristol-Myers Squibb (Personal fees)

### Acknowledgement

Our studies outlined in this paper were honored with the prestigious Medical Award of The Japan Medical Association. The author expresses his deepest appreciation to the many people who made this possible, including the following: the patients who kindly took part in the clinical trials; the members of all departments and basic research sections of the Keio University School of Medicine and Hospital; the Japan Clinical Oncology Group Data Center; the staffs of the institutions that participated in our studies, the multicenter study jointly conducted by the Japan Esophageal Society and the Japanese Gastric Cancer Association, and the multicenter trial organized by the Japanese Society for Sentinel Node Navigation Surgery; and the many experts who provided advice, especially the author’s mentors, Drs. Osahiko Abe and Masaki Kitajima.

### Disclaimer

Yuko Kitagawa is one of the Editors of JMA Journal and on the journal’s Editorial Staff. He was not involved in the editorial evaluation or decision to accept this article for publication at all.
